# Fulminant coronavirus disease 2019 meningitis in Iranian infants: a case series 

**DOI:** 10.1186/s13256-024-04428-z

**Published:** 2024-03-20

**Authors:** Shahla Afsharpayman, Sedigheh Madani, Susan Amirsalari, Nooradin Momeni, Mohammad Torkaman, Fatemeh Beiraghdar, Zohreh Kavehmanesh, Zahra Hosseininezhad

**Affiliations:** 1 Department of Pediatrics, Faculty of Medicine, Baqiyatallah University of Medical Sciences, Tehran, I. R. of Iran; 2 Fetal Health Research Center, Hope Generation Foundation, Tehran, I. R. of Iran

**Keywords:** COVID-19, Meningitis, Infant, Children, Case report

## Abstract

**Background:**

Pediatric coronavirus disease 2019 infection usually presents with respiratory and gastrointestinal symptoms. In this report we present fulminant meningitis as the main presentation of coronavirus disease 2019 without major signs and symptoms of other organs’ involvement in 3 infants.

**Cases:**

The first case was a 4 months Iranian male infant with fulminant meningitis as the main presentation of coronavirus disease 2019 without other organ involvement. He was treated as suspected bacterial meningitis but CSF PCR and CSF culture were negative for common meningeal pathogens. On 3rd day, his coronavirus disease 2019 PCR test became positive, while it was negative on 1st day. The second case was a 13 months Iranian male infant with fever, irritability, and photophobia for 24 h before poorly controlled status epilepticus. CSF coronavirus disease 2019 PCR became positive while CSF PCR and CSF culture were negative for other common meningeal pathogens. Seizures were controlled with multiple anti-seizure medications. The third case was a 14 months Iranian female infant with fever and seizure1 hour before admission, leading to poorly controlled status epilepticus despite anti-epileptic therapy 10 h after admission. CSF coronavirus disease 2019 PCR became positive while CSF PCR and CSF culture were negative for other common meningeal pathogens. He was controlled with multiple anti-seizure medications.

**Conclusion:**

Meningitis of coronavirus disease 2019 should be considered in severely ill pediatric cases with poorly controlled seizures and RBC in CSF smear. Also, pediatricians can consider corticosteroids, remdesivir, and IVIG therapy in these cases.

## Background

COVID-19 infection in children usually presents with respiratory and gastrointestinal symptoms. Searching PubMed with syntax (“COVID-19”[Mesh] AND “Meningitis”[Mesh]) resulted in 71 articles, out of which, 10 were adults and 4 were pediatric cases reported with COVID-19 meningo-encephalitis. Also, a systematic review in this regard mentioned 54 cases (mean age 50.8 ± 19 years) reported to have COVID-19 related meningo-encephalitis in which 3 cases had positive CSF PCR for COVID-19. This search result, beside loss of smell and taste sensation following olfactory nerve involvement, demonstrated COVID-19 neurotropic presentation [1–14].

In this report we present fulminant meningitis as the main presentation of COVID-19 without major signs and symptoms of other organs’ involvement in 3 infants.

## Case 1

An Iranian 4 months well-nourished male infant was referred to the emergency department with fever, poor feeding, and frequent vomiting. His symptoms appeared 12 h before admission and he was severely ill when admitted to PICU. Systemic physical examination showed fever (39 °C), pulse rate 120/min, blood pressure 80/50 mmHg, respiratory rate 34/min, hypotonia, sunken eyes, frequent vomiting despite not feeding recently. No respiratory symptoms were observed. Other physical examinations were normal and fontanels were not bulged. In the neurological examinations, the infant was restless and the cranial nerves examinations were normal. Muscles’ tone and force were normal and his deep tendon reflexes were in the normal range. His head circumference was 41.5 cm.

After parents gave written informed consent for procedures, sepsis laboratory work up was performed immediately (Table 1). Retinal fundoscopy revealed no edema. Lumbar puncture was done for Cerebrospinal Fluid (CSF) analysis (Table 1). Abnormal staring, oral automatism, and head colonic movements occurred2 hours after admission. IV phenytoin (20 mg/kg/stat then 2.5 mg/kg every 12 h) was started to prevent another attack of complex partial seizures.

**Table 1:** Laboratory documents of 3 cases during admission

Variables	At admission	During hospitalization	Before discharge
Case 1			
WBC (No.)	12,900	14,800	8400
PMN (%)	71	68	42
Lymphocyte (%)	23	24	57
Platelets (count No.)	325,000	405,000	435,000
ESR (mm/h)	26	105	7
CRP (mg/L)	70	64	5
CSF RBC (No.)	10		
CSF WBC (No.) (PMN%/lymphocyte%)	350 (20/80)		
CSF protein (mg/dL)	172		
CSF glucose (mg/dL)	76		
CSF gram stain	Negative		
CSF culture for bacteria	Negative		
CSF PCR for common bacterial germs of meningitis, HSV, and Candida	Negative		
Pharyngeal PCR for COVID-19	Positive		
CSF PCR for COVID-19	Not performed		
Blood culture (aerobes and anaerobes, and fungus)	Negative		
BUN (mg/dl)	13		
Creatinine (mg/dl)	0.3		
AST (IU/L)	23		
ALT (IU/L)	21		
Urine analysis	Normal		
Urine culture	Negative		
Case 2			
WBC (No.)	18,800	20,500	14,000
PMN (%)	68	81	39
Lymphocyte (%)	23	13	49
Platelets (count No.)	450,000	386,000	402,000
ESR (mm/h)	7	7	5
CRP (mg/L)	3.5	23	5
CSF RBC (No.)	100		
CSF WBC (No.) (PMN%/lymphocyte%)	400 (30/70)		
CSF protein	28		
CSF Glucose	145		
CSF gram stain	Negative		
CSF culture for bacteria	Negative		
CSF PCR for common bacterial germs of meningitis, HSV, and Candida	Negative		
Pharyngeal PCR for COVID-19	Positive		
CSF PCR for COVID-19	Popsitive		
Blood culture (aerobes and anaerobes, and fungus)	Negative		
BUN (mg/dl)	15		
Creatinine (mg/dl)	0.4		
AST (IU/L)	20		
ALT (IU/L)	23		
Urine analysis	Normal		
Urine culture	Negative		
Case 3			
WBC (No.)	14,300	11,000	4400
PMN (%)	71	65	39
Lymphocyte (%)	23	28	55
Platelets (count No.)	82,000	230,000	230,000
ESR (mm/h)	54	41	41
CRP (mg/L)	14	34	9
CSF RBC (No.)	110		
CSF WBC (No.) (PMN%/lymphocyte%)	0		
CSF Protein (mg/dL)	14		
CSF Glucose (mg/dL)	77		
CSF gram stain	Negative		
CSF culture for bacteria	Negative		
CSF PCR for common bacterial germs of meningitis, HSV, and Candida	Negative		
Pharyngeal PCR for COVID-19	Negative		
CSF PCR for COVID-19	Positive		
Blood culture (aerobes and anaerobes, and fungus)	Negative		
BUN (mg/dl)	10		
Creatinine (mg/dl)	0.5		
AST (IU/L)	19		
ALT (IU/L)	21		
Urine analysis	Normal		
Urine culture	Negative		

CSF and blood samples for aerobe, anaerobe, and fungus were collected and transported in sterile condition at admission time. Blood sample was obtained in sterile condition for common viral, bacterial, and fungal pathogens andPCR. CSF samples were obtained and transferred in sterile condition for COVID-19 infection test

*WBC* white blood cells, *PMN* Polymorph nuclear neutrophils, *ESR* Erythrocyte sedimentation rate, *BUN* Blood urea nitrogen, *AST* Aspartate aminotransferase, *ALT* Alanin aminotransferase, *PCR* polymerase chain reaction, *CRP* C-reactive protein

Important laboratory tests are presented in Table 1. CSF smear analysis was in favor of meningitis. Chest X ray illustrated disseminated fine reticular involvement of interstitial tissue of lungs and also cardiothoracic ratio enhancement. Cardiac consultation and echocardiography revealed normal cardiac function.

**Fig. 1:**
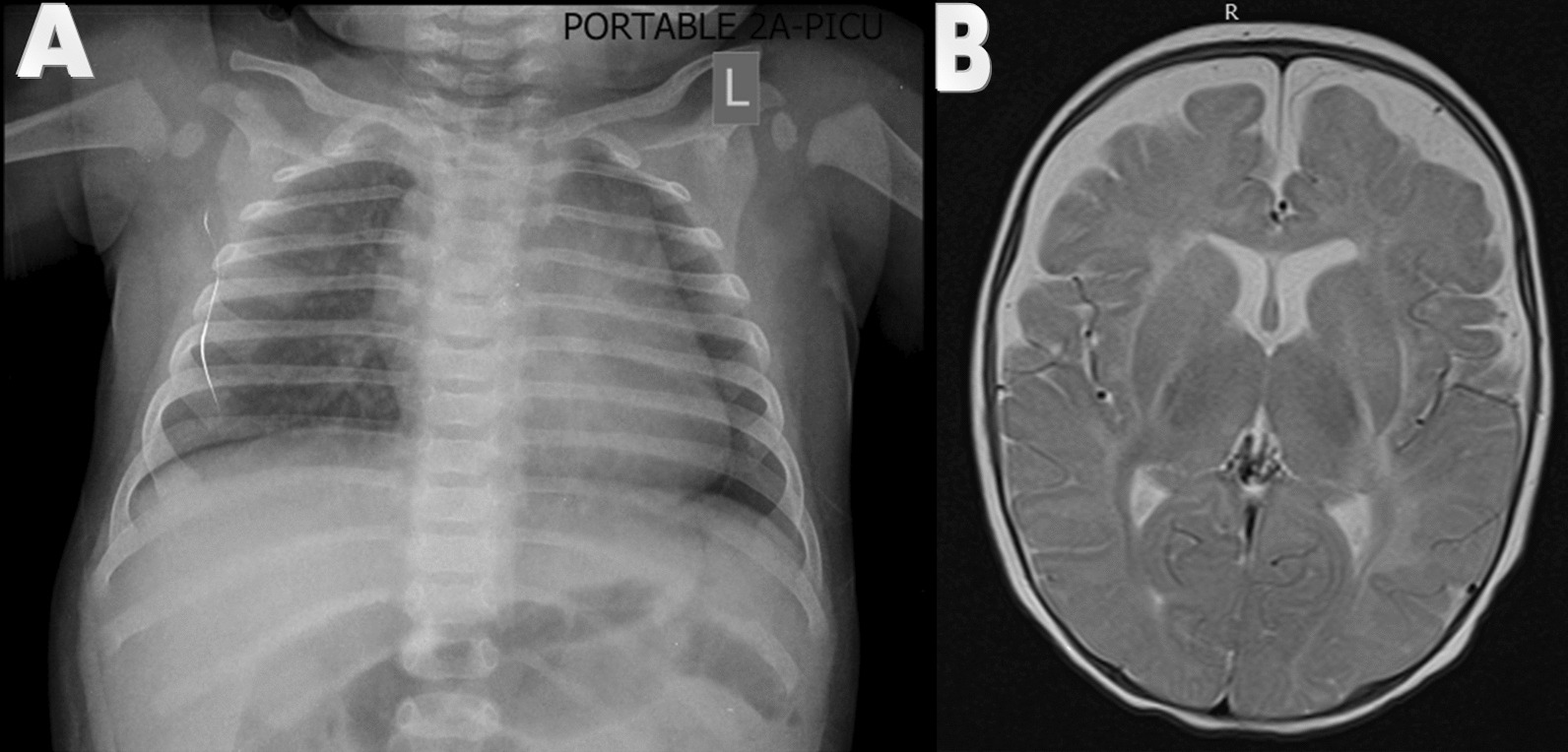
**A** Brain MRI of case 1 revealed Sub-Arachnoid fluid accumulation without hydrocephalus. **B** Chest X ray illustrated disseminated fine reticular involvement of interstitial tissue of lungs and cardiothoracic ratio enhancement

The patient was treated with intra venous (IV) cefotaxime (50 mg/kg every 6 h), IV vancomycin (15 mg/kg every 6 h), and 48 h IV dexamethasone (0.15 mg/kg every 6 h). Eight hours after admission he became hypothermic (35.5 °C) and his seizures recurred. IV immune globulin (IVIG) (1 gr/kg once), IV acyclovir (20 mg/kg every 8 h), and IV levetiracetam (30 mg/kg/stat then 15 mg/kg every 12 h) were added. Parents did not permit to take the second CSF sample. Thus, meningitis was treated with full anti-microbial and supportive treatments. Brain MRI revealed frontal sub-arachnoid CSF accumulation (Fig. 1). Serial head circumference measurement was performed and accelerated rate was not detected.

His general condition improved considerably and no fever was recorded after 2 days. However, on 3rd day he had clear coryza and mild diarrhea for one day. Infant’s second pharyngeal COVID-19 PCR was positive but CSF COVID-19PCR was not done.

CSF culture and CSF PCR were negative for common bacterial and viral pathogens of meningitis. In accordance to his good general condition and normal laboratory test son 6th day, anti-microbial therapy (cefotaxime, vancomycin, acyclovir) was discontinued. Phenytoin (2.5 mg/kg every 12 h) and levetiracetam (15 mg/kg every 12 h) were administered orally.

He was seizure free for 3 months after discharge and had normal neurological function and electro encephalogram (EEG). The oral phenytoin was then tapered off, and oral levetiracetam (15 mg/kg every 12 h) was continued. After 6 months of follow up, he was seizure free with normal neurological function and EEG and oral levetiracetam was tapered off.

## Case 2

An Iranian 13 months male infant was admitted in the emergency department with fever (38.5 °C), heart rate 110/min, respiratory rate 31/min, blood pressure 90/55 mmHg, poor feeding, vomiting, and photophobia from 6 h before admission. The infant's growth and development were normal but he had speech delay before recent sickness. No respiratory symptoms were observed. In the neurological examinations, the infant had irritable cries. Redor, Kernig’s, and Brudzinski’s signs were negative. Cranial nerves examinations were normal and muscles’ tone and force were normal. His deep tendon reflexes were in the normal range and the infant’s head circumference was 46.5 cm. Other physical examinations were normal.

**Fig. 2:**
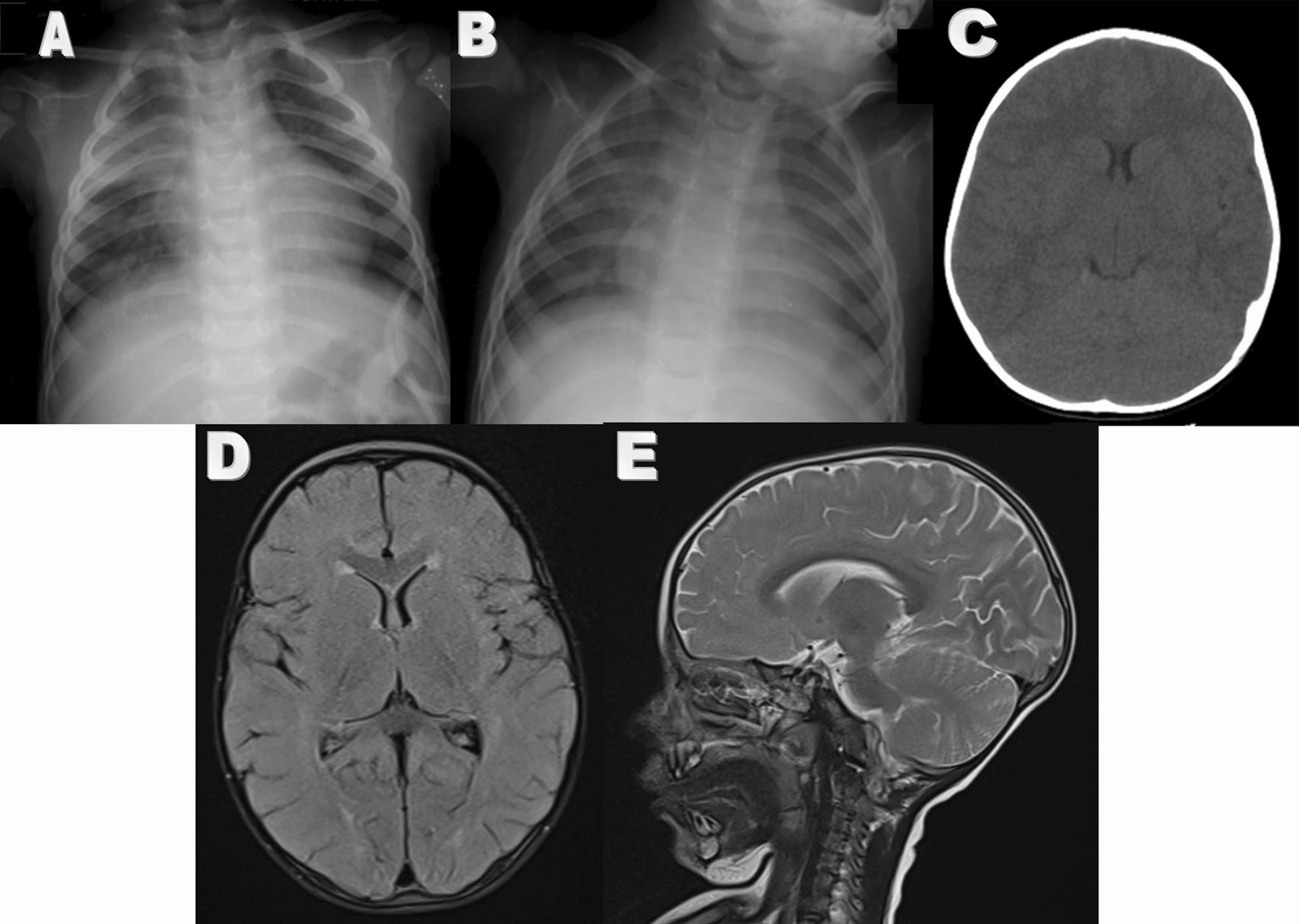
First chest X ray (**A**) illustrated right lung middle lob consolidation and the second one (**B**) was normal. Brain CT (**C**) and Brain MRI (**D**, **E**) of case 2

After parents gave written informed consent for procedures, sepsis laboratory work up was performed immediately (Table 1). Upward gaze and tonic-colonic movements occurred 12 h after admission. Diazepam (0.2 mg/kg/stat) was injected intravenously but the seizures continued. Therefore, IV Phenobarbital (20 mg/kg/stat) was administered but seizures recurred in an hour and prolonged for more than 10 min. To stop seizure, IV phenytoin (20 mg/kg/stat) then IV levetiracetam (30 mg/kg/stat) were loaded. After control of seizures, phenobarbital (2.5 mg/kg every 12 h) and levetiracetam (15 mg/kg every 12 h) were continued intravenously. Patient brain CT scan (Fig. 2) was normal. Inter-ictal EEG was normal. CSF analysis and other important laboratory tests results are shown in Table 1.

CSF analysis was in favor of meningitis. The patient received IV cefotaxime (50 mg/kg every 6 h), IV vancomycin (15 mg/kg every 6 h), and IV dexamethasone (0.15 mg/kg every 6 h). On the next day, patient was very hypotonic and also had poor sucking. He had some cough with normal respiratory rate (25/min) and oxygen saturation (> 94%). Chest X ray (Fig. 2) showed right lung middle lob consolidation. HisCOVID-19 PCR of pharyngeal and CSF samples became positive; while, CSF culture, and CSF PCR for common pathogens of meningitis and encephalitis were negative. So, IV cefotaxime (50 mg/kg every 6 h) and IV vancomycin (15 mg/kg every 6 h) were continued for aspiration pneumonia and IV acyclovir was discontinued. With considering COVID-19 infection, IV Dexamethasone (0.15 mg/kg every 6 h) was continued for 5 more days and IV remdesivir (first day 5 mg/kg/day and next 4 days 2.5 mg/kg/day) were added because of prolonged fever.

Cardiac consultation and echocardiography revealed normal cardiac function. Neurologic consultation was performed for his hypotonia, and neurologist recommended reducing IV phenobarbital (1.5 mg/kg every 12 h). Brain MRI was normal for age (Fig. 2). After 2 days, fever, hypotonia, and photophobia were resolved. A week after admission, some papular rash appeared on the stomach and thigh. Papular rash disappeared after stopping phenobarbital with hydrocortisone and cetirizine therapy. Clobazam (2.5 mg every 12 h) was added instead of phenobarbital. Chest X ray became clear after 12 days (Fig. 2). So, cefotaxime and vancomycin were discontinued after 14 days and infant was discharged with oral clobazam (2.5 mg every 12 h) and oral levetiracetam (15 mg/kg every 12 h). After 3 months, he was seizure free and had normal EEG, so anti seizure medications was discontinued gradually. After 6 months he was still seizure free and he was followed for his speech delay that he had before the meningitis.

## Case 3

A 14 months Iranian female infant with fever (39 °C) and seizure was admitted. She had normal growth and millstones. Her illness began with upper respiratory symptoms 4 days before admission. At emergency room, she received IV diazepam (0.2 mg/kg/stat) and after 5 min, IV phenytoin (20 mg/kg/stat then 2.5 mg/kg every 12 h) was loaded because of prolonged seizure. Then, patient became stable and her respiratory rate, pulse rate, and blood pressure were 28/min, 107/min, and 94/54 mmHg, respectively. Physical examinations were normal after postictal phase and the infant became alert. Neurological examinations such as Redor, Kernig’s, and Brudzinski’s signs, and cranial nerve functions were normal. Muscles’ tone and force were normal, and her deep tendon reflexes were in the normal range. Her head circumference was 47.5 cm.

**Fig. 3:**
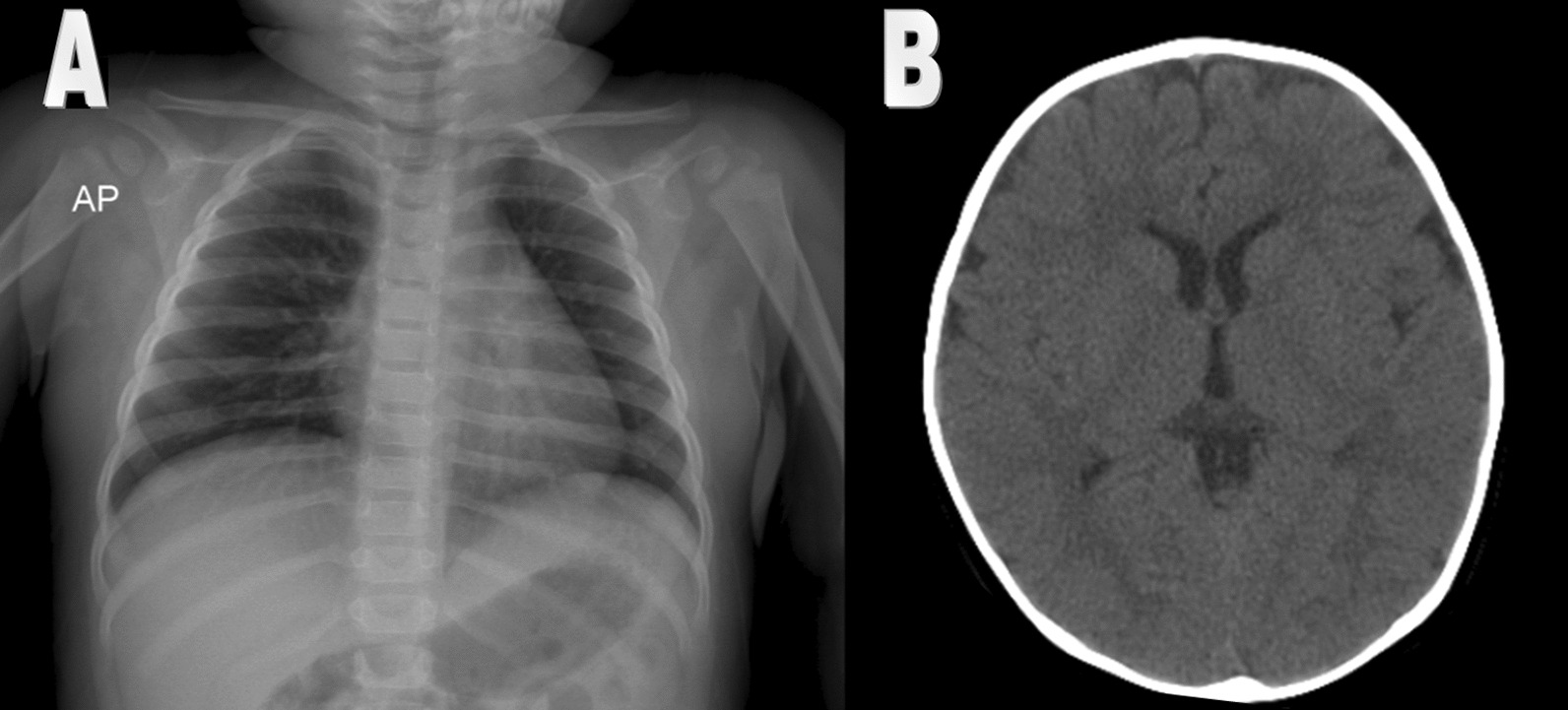
In case 3 chest X ray (**A**) revealed reticular changes and Brain CT scan (**B**) was normal

After 10 h of her first seizure, she developed another poorly controlled status epilepticus. Her second seizure lasted for 15 min and were finally controlled by one dose of IV diazepam (0.2 mg/kg/stat) and IV levetiracetam (30 mg/kg/stat then 15 mg/kg every 12 h). After parents gave written informed consent, additional procedures were performed. Her brain CT scan (Fig. 3) was normal and lumbar puncture had no WBC (Table 1). CXR revealed reticular involvement (Fig. 3) so IV cefotaxime (50 mg/kg/dose every 8 h) was administered for 7 days. Tests results are shown in Table 1. Pharyngeal COVID-19 PCR was negative while CSF COVID-19 PCR became positive, so, IV dexamethasone (0.15 mg/kg every 6 h) was administered for COVID-19 meningitis. CSF culture and CSF PCR for common pathogens of meningitis and encephalitis were negative. Fever resolved on the third day of admission and her general condition improved. She was discharged with oral levetiracetam (15 mg/kg every 12 h) after 7 days. She had normal EEG and was seizure free after 3 months. Therefore, oral levetiracetam was tapered off. During 6 months follow up, she had normal neurological function and normal EEG, without any episode of seizure.

## Discussion

Briefly, the first case was a 4 months male infant with fulminant meningitis as the main presentation of COVID-19 without other organ involvement. He was treated as suspected bacterial meningitis but CSF PCR and CSF culture were negative and on 3rd day, his pharyngeal COVID-19 PCR test became positive, while it was negative on 1st day. The second case was a 13 months male infant with fever, irritability and photophobia for 24 h before poorly controlled status epilepticus. CSF COVID-19 PCR became positive. Seizures were controlled with multiple anti-seizure medications. The third case was a 14 months female infant with fever and seizure1 hour before admission, leading to poorly controlled status epilepticus despite anti-epileptic therapy 10 h after admission. CSF COVID-19 PCR became positive. He was controlled with multiple anti-seizure medications. All infants improved without sequelae and their anti-seizure medications were discontinued after 3 or 6 months.

We present 3 infants with COVID-19 meningitis and poorly controlled seizures as their main primary manifestation. Despite severe presentations, their clinical condition improved after 2–4 days. Dexamethasone, besides remdesivir, or IVIG was administered immediately after seizures to cover fulminant meningitis and it seems they had an important role in improving patients’ condition.

COVID-19 infection has variety of clinical manifestations in pediatric cases and it mimics a lot of other viral and bacterial infections’ manifestations [15]. In addition, previous studies have mentioned considerable mortality and morbidity rate in Iranian infants infected with COVID-19 [16, 17]. To the best of our knowledge there were 2 reports in which an infant and a neonate had meningitis with positive CSF PCR for COVID-19 [1, 18]. In one of these articles, a 5 months old infant presented with the history of 4 days of COVID-19 general symptoms, then meningitis progressed and its general condition worsened until IVIG administration. In the other article, a neonate with poor feeding and lethargy had been worked up for sepsis and his general condition improved with supportive treatments.

Fulminant presentation is a medical indicator for any sudden and rapid progressive process. Fulminant meningitis was previously defined for some bacterial meningitis with rapid progressive meningeal inflammation that leads to severe illness and increased intracranial pressure. In our cases 1 and 2, symptomatic meningitis presented before COVID-19 general symptoms.

In cases 1 and 2, high percentage of lymphocyte and less PMN besides RBC was observed in CSF smear. This pattern has been seen in herpes meningitis and early phase of common bacterial meningitis. So, vancomycin, cefotaxime, and acyclovir were administered until CSF PCR and culture revealed that the source of meningitis was COVID-19. The third case, CSF smear had no WBC but it had 110 RBC. Therefore, in these 3 cases, the common feature was presence of RBC in CSF smear and two cases had CSF smear appeared like herpes meningitis. Preschool children, especially infants are at risk of several viral infections besides COVID-19. Laboratory changes of COVID-19 mimic different viral and bacterial patterns. For example in case1, both ESR and CRP values increased initially and then reduced with clinical improvement, but in case 2, the ESR remained unchanged and normal while a mild elevation was seen in CRP level after 3 days of admission. Relative lymphopenia has been considered as a mark for COVID-19 infection especially in pediatric cases and it had been seen in our cases, too.

Chest CT scan is the diagnostic imaging in adults who are infected by COVID-19, but in children is not recommended routinely because of risk of post X ray tumorigenicity [19]. In these cases, we did not perform chest CT scan because they had not prominent respiratory symptoms. This imaging modality can be considered in COVID-19 infected children who have poorly controlled respiratory symptoms.

Brain imaging such as MRI and CT may have some normal variation during infancy. Differentiation between pathologic changes and normal variations will be diagnosed by follow up brain imaging and neurological examinations. In cases 1 and 2, brain MRI was performed for their severe illness and lethargy besides poorly controlled seizures. Because of closed fontanels, brain CT scan was performed in cases 2 and 3, to rule out space occupying lesion and brain edema that could have caused brain hernia during lumbar puncture.

Pediatricians should consider COVID-19 as a potential infection that could have serious manifestations such as rapidly progressive meningitis and start corticosteroid, remdesivir, and IVIG as potential treatments.

## Conclusion

To the best of our knowledge, there is one report of a neonate with COVID-19 meningitis with positive CSF PCR for COVID-19 and here we add 3 more positive CSF PCR for COVID-19 infants in this article. Meningitis of COVID-19 should be considered in severely ill pediatric cases with poorly controlled seizures and RBC in CSF smear. Also, pediatricians can consider corticosteroids, remdesivir, and IVIG therapy in these cases.

## Data Availability

The data supporting this study finding can be presented by corresponding author if it is needed.

## Declarations

### Limitation

Lack of enough CSF sample for investigating COVID-19 as meningitis source.
